# Characteristics and risk factors of Health-Related Risky behaviors in adolescents with Depression

**DOI:** 10.1186/s13034-024-00722-2

**Published:** 2024-03-18

**Authors:** Hui Wang, Zhixiong Wang, Xue Li, Jing Liu

**Affiliations:** 1 Institute of Mental Health, Peking University Sixth Hospital, National Clinical Research Center for Mental Disorders (Peking University Sixth Hospital), Beijing, China; 2 Peking University HuiLongGuan Clinical Medical School, Bejing Huilongguan Hospital, Bejing, China

**Keywords:** Depression, Adolescent, Health-related risky behaviors, Related factors, Risk factors

## Abstract

**Backgound:**

To explore the characteristics and risk factors for health-related risky behaviours (HRRBs) in adolescents with depression.

**Methods:**

A total of 136 adolescents aged 12–18 years who met the diagnostic criteria for depression, and 272 healthy controls. All the subjects were assessed with the Adolescent Health-Related Risky Behavior Inventory (AHRBI), and the AHRBI scores of the two groups were compared with the Mann–Whitney U test. The depression group was assessed with the Self-Rating Anxiety Scale (SAS), Self-Rating Depression Scale (SDS), Childhood Trauma Questionnaire (CTQ), Cognitive Emotion Regulation Questionnaire (CERQ), Egna Minnen av Barndoms Uppfostran (EMBU), and Family Adaptability and Cohesion Scale (FACES II-CV). Spearman correlation analysis and multiple linear regression were used to explore the risk factors for HRRBs in adolescents with depression.

**Results:**

The AHRBI total score and five-factor scores of self-injury and suicide (SS), aggression and violence (AV), rule-breaking (RB), smoking and drinking (SD), and health-compromising behavior (HCB) in the depression group were higher than those in the control group. The severity of anxiety, catastrophizing, cognitive emotional regulation strategy (self-blame and blaming of others), the frequency of depression, physical neglect, and sexual abuse all increased the risk of HRRBs in adolescents with depression, and paternal emotional warmth and understanding had protective effects.

**Conclusion:**

First, depressed adolescents exhibited significantly more HRRBs than healthy adolescents. Second, there are many risk factors for HRRBs in adolescents with depression, and the risk factors for different types of HRRBs are also different.

## Background

Depression is the most common mood disorder, often leading patients to experience reduced interest and happiness, cognition dysfunction, and physical symptoms [1]; these symptoms can lead to dangerous behaviors (such as SS and AV) and functional impairment (such as academic burnout and lack of exercise) [2–4]. HRRBs refer to behaviors that have adverse effects on health, mainly including SS, AV, RB, SD, unprotected sexual behavior (USB), and HCB characterized by excessive food intake and inactivity [5]. According to current research and clinical observations, there exist distinct differences in clinical characteristics between adolescent depression and adult depression. Firstly, adolescent depression tends to manifest in a more diverse and intricate manner. Adolescents may exhibit more pronounced emotional fluctuations, encompassing extremes of elation and despondency, as well as rapid shifts in mood of shorter duration. Additionally, adolescents often internalize their emotional expressions, converting depressive feelings into physical symptoms such as headaches, stomachaches, rather than directly expressing feelings of low mood [6].Compared with other age groups, adolescents are more prone to HRRBs. In addition, patients with depression have significantly higher risks of various HRRBs due to depression symptoms. Therefore, it is necessary to pay attention to the HRRBs of adolescents with depression.

To date, research on HRRBs in depression patients has mainly focused on adults, and comprehensive and systematic research on HRRBs in adolescents with depression is lacking [7]. After reviewing the previous studies on HRRBs of adolescents with depression, we concluded that most focused on the dimensions of SS or AV, while there is a lack of exploration of other common HRRBs [8–10]. In addition, the definition of depression in these studies was vague, such as depressive mood, depressive state, depressive symptoms, depression that constitutes clinical diagnosis, and depressive disorders (in the above study) [11–13]. Given these gaps in the literature, there is a need to conduct a comprehensive and systematic analysis to describe the characteristics of HRRBs in adolescents with depression.

Because HRRBs have many adverse consequences for adolescent health, it is extremely important to identify the risk factors for HRRBs in adolescents with depression in addition to achieving a comprehensive understanding of the characteristics of HRRBs in adolescents with depression. In this regard, previous studies have focused on the factors influencing adolescent depression [14, 15], factors influencing individual risk behaviors (such as SS) [16, 17], or factors influencing risk behaviors in healthy adolescents [18], rather than the factors influencing various HRRBs in adolescents with depression. Moreover, relatively few risk factors have been included in previous analyses, and these factors have been limited in terms of demographic data, clinical characteristics, or use of a certain psychological test, etc. To address this gap, the present study aims to explore the risk factors for HRRBs in adolescents with depression in a more comprehensive manner, as these findings will provide direction and a basis for the prevention of HRRBs for at-risk populations.

In this study, the Adolescent Health-Related Risky Behavior Inventory (AHRBI) [19] was used to expansively investigate the characteristics of HRRBs in adolescents with depression along six different dimensions: SS, AV, RB, SD, HCB, and unprotected sexual behaviour (USB). In addition to demographic data, individual and environmental factors included clinical characteristics, childhood trauma exposure, cognitive emotional regulation strategy, parenting style, family adaptability, and cohesion to comprehensively discuss risk factors. The objective of the current study is to offer significant and insightful observations into HRRBs among adolescents with depression. Furthermore, it aims to provide indications for future research regarding relevant interventions, with the ultimate goal of enhancing the mental well-being of children and adolescents.

## Methods

### Participants

The depression group was adolescents with depression who were treated in the outpatient department and ward of Peking University Sixth Hospital from November 2018 to August 2019. The inclusion criteria for patients were as follows: ① diagnosed with depression (first depressive episode, recurrent depression, or bipolar depression) according to the International Statistical Classification of Diseases and Related Health Problems, 10th revision (ICD-10), by two qualified pediatric psychiatrists; ② aged 12 ∼ 18 years; and ③ able to understand and cooperate with questionnaire evaluation. The exclusion criteria were as follows: ① presence of comorbid mental disorder(s); ② history of severe somatic disease, nervous system disease, or brain injury; or ③ inability to cooperate to complete the assessment. A total of 141 patients were included in the study, of whom 5 were excluded due to incomplete data. Thus, a total of 136 patients (33 males and 103 females) were included in the study. The age range was 12 ∼ 18 years, with a median age of 16 years.

The control group consisted of students who were randomly selected from a middle school. The inclusion criteria for healthy controls (HCs) were as follows: ① did not meet any diagnostic criteria for mental disorders according to the ICD-10; ② aged 12 ∼ 18 years; and ③ able to understand and cooperate with the questionnaire evaluation. The exclusion criteria were as follows: a history of severe physical diseases, nervous system diseases, and brain trauma. To match the HCs according to the sex and age of the depression group, 300 individuals were randomly selected in a ratio of 1:2. Among them, 28 individuals were excluded due to invalid evaluation results. A total of 272 HCs (77 males and 195 females) were finally included in the control group. These HCs were aged 12–18 years, with a median age of 16 years.

There was no significant difference in age (Z=-0.049, *P* = 0.961) or sex (χ^2^ = 3.53, *P = 0.206)* between the depression group and the control group.

This study was approved by the Ethics Committee of Peking University Sixth Hospital (No. 2016-11-7-1). The subjects and their guardians understood the content and purpose of the study, agreed to participate in this study, and signed the informed consent form.

### Measures

#### General information record sheet

This part of the questionnaire consisted of custom-designed questions. According to the clinical interview and previous medical records, the researchers obtained the demographic data and clinical characteristics of the patients, including sex, age, family socioeconomic status, academic achievement, parental education level, onset age, total course of the disease, frequency of depressive episodes, severity of depression (mild, moderate and severe), psychotic symptoms, antidepressant treatment, family history of mental diseases, and presence of hypomania/manic episodes.

#### Adolescent health-related risky behaviour inventory (AHRBI)

This is a self-report inventory, which used to assess the HRRBs of adolescents [19]. There are 6 subscales: self-injury and suicide (SS), aggression and violence (AV), rule-breaking (RB), smoking and drinking (SD), health-compromising behavior (HCB), and unprotected sexual behavior (USB), consisting of 38 items. Each item is scored on a scale from 1 (never) to 5 (often); using these item scores, the scores of subjects on various types of HRRBs are calculated. Higher scores represent a more severe exhibition of HRRB. In this study, the scale was used to evaluate the HRRBs of all subjects within one year. For patients with less than one year’s course of disease in the depression group, risky behaviors were evaluated after depression onset.

#### Self-rating anxiety scale (SAS)

This scale was developed by W. K. Zung in 1971 [20]. It is a self-rating scale and can be used to assess the severity of anxiety symptoms. It consists of 20 items that reflect subjective feelings of anxiety. Each item is scored based on the frequency of symptom occurrence (on a scale from 1 to 4), with higher scores correlating with more severe symptoms of anxiety.

#### Self-rating depression scale (SDS)

Developed by W. K. Zung in 1965 [21], this is a self-rating scale that evaluates the severity of depressive symptoms. The scale contains 20 items reflecting subjective feelings of depression. Each item is scored on a four-point scale according to the frequency of symptoms, with higher scores indicating increased severity of depressive symptoms.

#### Cognitive emotion regulation questionnaire (CERQ)

Compiled by Garnefski [22], this questionnaire is used to assess the degree to which subjects adopt various emotional regulation strategies after encountering negative life events from a cognitive perspective. The scale includes 9 dimensions: self-blame, blaming of others, rumination, catastrophizing, acceptance, positive reappraisal, planning, positive refocusing, and putting into perspective, in which higher scores on certain dimensions reflect a higher level of adoption for this particular cognitive-emotional regulation strategy.

#### Childhood trauma questionnaire (CTQ)

Developed by the American clinical psychologist Bernstein (1998), this questionnaire is used to evaluate childhood traumatic experiences and includes five subscales: emotional abuse, physical abuse, sexual abuse, emotional neglect, and physical neglect. The version adopted in this study is the Chinese version [23], which consists of 28 items, including 25 clinical items and 3 validity items. This self-report scale is completed by participants, with items scored on a five-point scale. Higher participant scores on various trauma dimensions illustrate more severe trauma experienced in childhood.

#### Egna minnen av barndoms uppfostran (EMBU)

Compiled by Swedish clinical psychologist Perris et al. (1980), this scale is used to measure parental ideas, behaviors, and emotional expressions toward children. This study adopted the Chinese version [24], this measure contains 66 items, evaluated by children on a four-point scale; higher scores on each dimension signify increased exhibition of the associated parenting style. The paternal subscale contains six dimensions: emotional warmth and understanding, punishment and severity, excessive interference, preference for subjects, refusal and denial, and excessive protection. The maternal subscale contains five dimensions: emotional warmth and understanding, severe punishment, excessive interference, and preference for subjects’ refusal and denial.

#### Family adaptability and cohesion evaluation scales II - computer version (FACES II-CV)

Compiled by Olson (1982), this scale is used to measure the adaptability of family members to changes in rules at different stages of development and in various family situations (i.e., family adaptability) as well as the closeness of emotional ties between family members (i.e., family intimacy). The Chinese version was adopted in this study [25], which contains 58 items in this self-report evaluation, with each item scored on a five-point scale to determine the intimacy and adaptability of the subject’s family.

### Data analysis

Statistical analysis and figure generation were carried out in SPSS 20.0. The Kolmogorov‒Smirnov test was used to explore the normality of all measurement data and HRRB data, and variables conforming to a normal distribution are described with the mean ± standard deviation. An independent-sample t-test was used for comparisons between the two groups. Variables that did not conform to a normal distribution are described by the median (minimum and maximum), and the Mann‒Whitney U test was used for comparison between the two groups. Frequency data are described according to counts and percentages, and group comparisons were carried out by the χ^2^ test. All tests were two-tailed, and a *P* < 0.05 was considered statistically significant.

The AHRBI total score and each factor score in the depression group were not normally distributed (*P* < 0.05). Therefore, the nonparametric test (Mann‒Whitney U test) was used to compare the AHRBI total score, factor scores, and item scores between the depression group and the control group. Spearman correlation analysis was used to analyze the correlations of the AHRBI total scores and factor scores with demographic variables and SDS scores, which were significantly different between the depression group and control group, and to determine the factors related to HRRBs in adolescents with depression. Then, the AHRBI total score and factor scores, which significantly differed between the depression group and the control group, were used as dependent variables, and all variables in the Spearman correlation analyses that had a significant correlation with the AHRBI total score or factor scores were used as independent variables. All variables were entered simultaneously in the multiple linear regression analysis. We thus explored the risk factors for adolescent HRRBs.

## Results

### Comparison of HRRBs between the depression and control groups

The AHRBI total score in the depression group was higher than that in the control group (Z=-10.65, *P* < 0.05), and the scores of five AHRBI factors (SS, AV, RB, SD, and HCB) were also higher than those in the control group (Z=-3.27∼-14.21, *P* < 0.05). Among the 38 AHRBI items, 22 exhibited higher scores in the depression group than in the control group (Z=-14.91∼-2.10, all *P* < 0.05). There were no significant group differences in the scores on other items (Z=-1.850∼-0.502, all *P* > 0.05). See Table 4.1 for details.

### Correlations between AHRBI scores and scores of socio‑demographic data, SAS, SDS, CERQ, CTQ, EMBU, and FACES II-CV in the depression group

To explore the factors related to HRRBs that differed between the depression group and control group, scores on the AHRBI and SS, AV, RB, HCB, and SD factors were significantly higher in the depression group than in the control group, and demographic characteristics, clinical characteristics, SAS scores, SDS scores, family adaptability and cohesion, parenting style, cognitive emotional regulation strategy use, and childhood trauma were correlated with HRRBs. Based on the correlation coefficient, associations were labeled as strong (*r* > 0.6), moderate (0.3 < *r* ≤ 0.6), or weak (0.1 < *r* ≤ 0.3) (Akoglu, 2018). Specific results are as follows.

The AHRBI total score was strongly correlated with the SAS total score (*r* = 0.682). The AHRBI total score was moderately correlated with SDS score, family intimacy, family adaptability, maternal excessive interference, maternal refusal and denial, maternal severe punishment, self-blame, catastrophizing, emotional abuse, emotional neglect, physical neglect, and total CTQ score (*r*=-0.390 ∼ 0.557, all *P* < 0.05). The AHRBI total score was weakly correlated with paternal emotional warmth and understanding, maternal emotional warmth and understanding, paternal severe punishment, paternal preference for subjects, paternal refusal and denial, putting into perspective, paternal overprotection, blaming of others, rumination, positive reappraisal, positive reappraisal, physical abuse and sexual abuse (*r*=-0.279 ∼ 0.297, all P *< 0.05*). See Table 4.2 for details.

SS was strongly correlated with the SAS total score (*r* = 0.672) and SDS total score (*r* = 0.629). SS was moderately correlated with family intimacy, family adaptability, maternal excessive interference, catastrophizing, self-blame, rumination, positive refocusing, positive reappraisal, emotional abuse, emotional neglect, physical neglect, and the total CTQ score (*r*=-0.393 ∼ 0.527, all *P* < 0.0). SS was weakly associated with sex, depression severity, paternal emotional warmth and understanding, maternal emotional warmth and understanding, maternal severe punishment, paternal refusal and denial, maternal refusal and denial, paternal preference for subjects, maternal preference for subjects, putting into perspective, physical abuse, and sexual abuse (*r*=-0.269 ∼ 0.289, all P *< 0.05*). See Table 4.2 for details.

AV was not strongly associated with any factors. Factors moderately associated with AV were as follows: the total SAS score, paternal severe punishment, maternal excessive interference, paternal refusal and denial, catastrophizing, blaming of others, emotional abuse, emotional neglect, physical neglect, and the total CTQ score (*r* = 0.300 ∼ 0.407, all P *< 0.05*). Factors weakly related to AV were as follows: the total SDS score, family intimacy, family adaptability, paternal emotional warmth and understanding, maternal severe punishment, paternal excessive interference, maternal refusal and denial, paternal excessive protection, putting into perspective, physical abuse, and sexual abuse (*r*=-0.292 ∼ 0.298, all P *< 0.05*). See Table 4.2 for details.

RB was not strongly associated with any factors. Factors moderately related to RB were as follows: the total SAS score, physical neglect, and the total CTQ score (*r* = 0.303 ∼ 0.332, all P < *0.05*). The factors weakly related to RB were as follows: academic achievement, the total SDS score, a family history of mental disorder, family intimacy, family adaptability, paternal severe punishment, maternal excessive interference, maternal severe punishment, paternal refusal and denial, maternal refusal and denial, paternal overprotection, self-blame, catastrophizing, putting into perspective, emotional abuse, emotional neglect, and physical abuse (*r*=-0.280 ∼ 0.288, all P *< 0.05*). See Table 4.2 for details.

There were no factors strongly related to SD. Factors moderately related to SD were as follows: the total SAS score (*r* = 0.336) and catastrophizing (*r* = 0.367). Factors weakly related to SD were as follows: the total SDS score, family intimacy, maternal severe punishment, maternal excessive interference, paternal refusal and denial, maternal refusal and denial, paternal overprotection, blaming of others, self-blame, putting into perspective, emotional abuse, physical neglect, and the total CTQ score (*r*=-0.199 ∼ 0.278, all P *< 0.05*). See Table 4.2 for details.

The factors strongly related to HCB were the total SAS score (*r* = 0.625) and total SDS score (*r* = 0.603). Factors moderately related to HCB were family intimacy, family adaptability, paternal emotional warmth and understanding, positive reappraisal, catastrophizing, emotional abuse, emotional neglect, physical neglect, and the total CTQ score (*r*=-0.422 ∼ 0.448, all P *< 0.05*). Factors weakly related to HCB were sex, frequency of depressive episodes, maternal emotional warmth and understanding, paternal severe punishment, maternal excessive interference, maternal refusal and denial, maternal severe punishment, self-blame, rumination, positive reappraisal, and putting into perspective (*r*=-0.296 ∼ 0.288, all P *< 0.05*). See Table 4.2 for details.

### Multiple regression analysis of risk factors for HRRBs in the depression group

The AHRBI total score and five-factor scores, which significantly differed between the depression group and the control group, were used as the outcome variables, and all the variables significantly related to these variables in the Spearman correlation analyses were included as the predictive variables. Linear regression analysis was carried out by the enter method (i.e., simultaneous inclusion of all predictors). The results were as follows.

The SAS total score, catastrophizing, and maternal emotional warmth and understanding significantly predicted the AHRBI total score of adolescents with depression. The SAS score, self-blame, sexual abuse, and catastrophizing significantly predicted the SS of adolescents with depression. Family intimacy, family adaptability, blaming of others, and physical neglect significantly predicted AV in adolescents with depression. The frequency of depressive episodes, paternal emotional warmth and understanding, maternal emotional warmth and understanding, the SAS score and catastrophizing significantly predicted the HCB of adolescents with depression (all P *< 0.05*). No variables significantly predicted RB or SD of adolescents with depression (all P *> 0.05*). See Table 4.3, 4.4, 4.5, and 4.6 for details.

## Discussion

### HRRBs of adolescents with depression

This study comprehensively investigated the characteristics of HRRBs in adolescents with depression and found that the depression group exhibited more HRRBs than the control group, including SS, AV, RB, SD, and HCB, which was consistent with previous research findings. Previous studies have shown that adolescents with depression exhibit more SS, AV, truancy and unexplained absences, and SD than healthy adolescents [2, 7, 26, 27]. In terms of HCB, Hou Fangli and others found that the intake of “traditional food” by adolescents with depressive symptoms decreased, while the intake of “snacks” and “high energy” food increased [28]. Some studies have also found that depressive symptoms positively predict sedentary behavior in female adolescents [29].

The increased HRRBs in adolescents with depression are thought to be related to the clinical characteristics of adolescents with depression. For example, SS and irritability are common clinical symptoms of depression in adolescents [30]. Frequent irritability can cause more aggression and violent impulses in adolescents with depression [31]. In terms of RB, adolescents with depression lack energy, interest and motivation, which can make it difficult for them to go to school [32]. If people around adolescents with depression have conflicts with them due to academic problems, which will lead to more RB, such as truancy, unexplained absences, and running away from home. In terms of SD, previous studies have also shown that adolescents with depression tend to drink to help them sleep and reduce their perceived depression and tend to smoke to alleviate anxiety and uneasiness [33]. The HCB of adolescents with depression is also considered to be related to clinical characteristics, such as loss of appetite, sleep disorders, and lack of energy.

### Risk factors for HRRBs in adolescents with depression

#### Overall HRRBs

This study explored the risk factors for overall HRRBs and found that anxiety was a risk factor for HRRBs in adolescents with depression. Previous studies also found that risky behaviors may be related to increased severity of symptoms of anxiety and depression [34, 35]. In terms of individual factors, this study showed that catastrophizing (a cognitive emotional regulation strategy) positively predicted the overall HRRBs of adolescents with depression. At present, no research has focused on the correlation between cognitive emotional regulation style and the overall HRRBs of adolescents with depression, but some studies have found that catastrophizing can predict adolescent SS and aggressive behavior [36]. Catastrophizing further aggravates the negative feelings associated with depression, such as pessimism or sensitivity and paranoia, which leads to more SS or impulsive aggression [37]. In terms of environmental factors, we found that maternal emotional warmth and understanding was a risk factor for the overall HRRBs of adolescents with depression, which is somewhat counterintuitive. However, excessive warmth and understanding (e.g., permissive parenting styles) may lead to insufficient guidance. Moreover, previous studies have shown that if parents set specific behavioral rules, their children exhibit fewer risky behaviors than observed in response to general parental support and discipline [38], which indicates that maternal warmth and understanding must be within a moderate range to promote children’s development.

### SS

We found that the SAS score was a risk factor for SS in adolescents with depression, similar to previous studies. Some studies have even found that SAS scores can distinguish between individuals with and without suicidal behavior [39]. Some studies have suggested that anxiety symptoms in depression indicate weaker resilience and emotional regulation [40], which could partially explain the increased risk of SS. We also found that two negative cognitive emotional regulation strategies (i.e., self-blame and catastrophizing) were risk factors for SS in adolescents with depression. Previously, researchers divided adolescent behavior into internalizing problems (such as SS) and externalizing problems (such as AV) and found that self-blame was an independent predictor of internalizing problems [36], which suggests that researchers and healthcare providers should pay more attention to negative thought patterns in individuals when managing the risk of SS. In terms of environmental factors, it was found that sexual abuse was a risk factor for SS in adolescents with depression. LeMoult conducted a meta-analysis and found that childhood sexual abuse not only increased the risk of depression but also increased the risk of posttraumatic stress disorder, suicide, poor academic performance, and promiscuity [41], which supports the results of the present paper.

### AV

It was found that blaming others was a risk factor for AV in adolescents with depression. Previous studies have found that total scores of anger, hostility, and aggression were significantly higher in depressive patients than in the control group. Studies have found that when adolescents with depression use the cognitive emotional regulation strategy of blaming others, anger and hostility toward the outside world lead to AV [36, 42]. In terms of environmental factors, family adaptability (the ability of the family system to change in response to the environment and in response to problems at different stages of development) was a risk factor for adolescents with depression in our study. However, previous studies have shown that good family adaptability can reduce the risk of AV in healthy adolescents [43]. This discrepancy may be related to the different subject pools.

### HCBs

In terms of disease factors, we found that the frequency of depressive episodes and the SAS score were risk factors for HCB in adolescents with depression. This result is thought to be due to the physical influence of depression and anxiety. In terms of individual factors, we found that catastrophizing is a risk factor for HCB in adolescents with depression. Although similar studies did not reach this conclusion, some have shown that the more intense the catastrophizing behavior, the more distressed adults feel [44], which indicates that catastrophizing aggravates patients’ physical discomfort, resulting in HCB. Some studies have also shown that catastrophizing increases people’s fear of injury and avoidance of exercise [45]. In terms of environmental factors, we found that maternal emotional warmth and understanding was a risk factor for HCB in adolescents with depression. Although there is no previous research on this topic, in the clinic, we have observed that mothers with excessive emotional warmth and understanding are often more anxious, which may lead to excessive supervision that leads to rebellious behavior in children. In this study, paternal emotional warmth and understanding was a protective factor against HCB in adolescents with depression. No similar studies have been conducted, but some studies have shown that children from single-mother families are more likely to exhibit risky behavior in adolescence than those from two-parent families [46], which indicates the importance of the paternal parenting role. In China, it is relatively more common to have a distribution of family functional roles where the father is responsible for earning money, while the mother takes charge of the children. However, this arrangement can lead to reduced communication between the father and child. The results of our study suggest that moderately increasing paternal emotional warmth and understanding and reducing maternal “control” may reduce the occurrence of HCB in adolescents with depression.

In this study, no variables were found to significantly predict the RB or SD of adolescents with depression. However, previous studies have shown that the frequency of depressive episodes and family conflicts increases the risk of substance abuse among adolescents [47, 48]. The negative results of this study may be due to its small sample size.

### Limitations and future directions

As the sample size was relatively small within the current study, the generalizability of findings may be limited. Moreover, the cross-sectional design of the study precludes any causal inferences being made from follow-up data. Future studies should continuously evaluate HRRBs and their risk factors before and after illness and treatment in adolescents with depression to provide a more in-depth and comprehensive understanding of changing HRRB risk factors over time.

The findings of this study have several important implications. Notably, the results highlight the multifaceted nature of risk factors for HRRBs in adolescents with depression, encompassing disease-related, individual, and environmental aspects. This underscores the need for comprehensive, multi-dimensional approaches in the prevention and treatment of HRRBs in this population. For instance, interventions could be designed to target not only the symptoms of depression and anxiety but also maladaptive cognitive emotional regulation strategies such as catastrophizing and self-blame.

Furthermore, the finding that maternal emotional warmth and understanding was a risk factor for HRRBs, while paternal emotional warmth and understanding was a protective factor, is somewhat counterintuitive. This suggests that the roles and influences of mothers and fathers may differ in the context of adolescent depression and HRRBs, and this warrants further investigation. It also implies that family-based interventions may need to consider the differential roles of mothers and fathers and aim to promote a balanced parenting approach.

The findings of this study underscore the complexity of HRRBs in adolescents with depression and highlight the need for further research and comprehensive, tailored interventions to effectively open new opportunities and avenues for successful, positive intervention.

## Conclusion

The overall HRRBs, AV, RB, SS, SD, and HCB of adolescents with depression were significantly higher than those of healthy adolescents. There are many risk factors for HRRBs in adolescents with depression. Except for SD and RB, the risk factors for different HRRBs are different, involving all aspects of disease, the individual, and the environment. Factors such as the severity of anxiety, catastrophizing, cognitive-emotional regulation strategies including self-blame and blaming others, the frequency of depressive episodes, neglect of physical health, and experiences of sexual abuse significantly elevate the risk of engaging in health-related risky behaviors (HRRBs) among adolescents experiencing depressive episodes. Conversely, a high degree of family intimacy and paternal emotional warmth and understanding offer protective effects. The study aims to further increase the sample size, include a broader range of influencing factors, and dynamically evaluate the fluctuation of various HRRBs in adolescents undergoing depressive episodes across different stages of the condition, thereby establishing a foundation for preventative measures and therapeutic interventions.

## Data Availability

No datasets were generated or analysed during the current study.

## Appendix

**Table 4.1:** Comparison of AHRBI scores between the adolescent depression group and control group [median (minimum, maximum)]

Factor/item		Depression group (*n* = 136)		Control group (*n* = 272)		Z		P
**AHRBI total score**		63.5 (44, 121)		49 (42, 87)		-10.65		<0.001
Truancy or absence		1 (1, 5)		1 (1, 3)		-9.21		<0.001
Running away from home		1 (1, 4)		1 (1, 3)		-8.54		<0.001
Lying to family members		3 (1, 5)		2 (1, 4)		-5.10		<0.001
Stealing money		1 (1, 5)		1 (1, 3)		-5.54		<0.001
**Suicide and self-injury behavior**		13 (5, 21)		5 (5, 15)		-14.21		<0.001
Trying to cut or burn yourself		3 (1, 5)		1 (1, 4)		-11.74		<0.001
Having suicidal thoughts		3 (1, 5)		1 (1, 4)		-14.91		<0.001
Hurting yourself by biting, scratching, etc.		3 (1, 5)		1 (1, 4)		-10.39		<0.001
Planning suicide		1 (1, 5)		1 (1, 3)		-3.18		0.001
Attempted to commit suicide		2 (1, 5)		1 (1, 3)		-13.34		<0.001
**Health-compromising behavior**		12 (5, 25)		10 (5, 17)		-6.46		<0.001
Does not have breakfast		4 (1, 5)		5 (2, 5)		-8.46		<0.001
Does not drink milk/soy milk		4 (1, 5)		4 (1, 5)		-2.10		0.036
Does not participate in sports activities		3 (1, 5)		1 (1, 5)		-8.12		<0.001
Overeating or vomiting		3 (1, 5)		1 (1, 3)		-14.04		<0.001
Excessive dieting		1 (1, 5)		1 (1, 4)		-5.28		<0.001
**Aggression and violent behavior**		15 (10, 35)		13 (10, 27)		-5.49		<0.001
Bullying, threatening or intimidating a companion		1 (1, 5)		1 (1, 3)		-4.58		<0.001
Intentional injury to others		1 (1, 5)		1 (1, 4)		-4.16		<0.001
Retaliatory action		2 (1, 5)		1 (1, 4)		-6.36		<0.001
Robbing		1 (1, 5)		1 (1, 3)		-4.07		<0.001
Carrying a weapon		1 (1, 5)		1 (1, 3)		-7.35		<0.001
**Smoking and drinking behavior**		6 (6, 24)		6 (6, 19)		-3.27		0.001
Smoking		1 (1, 5)		1 (1, 4)		-3.71		<0.001
Peer pressure to smoke		1 (1, 5)		1 (1, 4)		-2.80		0.005
**Unprotected sexual behavior**	5 (5, 9)		5 (5, 15)		-0.92		0.356	
**Rule-breaking behavior**		11 (7, 22)		9 (7, 17)		-7.81		<0.001

**Table 4.2:** Analysis of factors related to HRRBs in adolescents with depression

	Self-injuring and suicide	Aggression and violent behavior		Health-compromising behavior	Smoking and drinking	AHRBI total score
**Demographic characteristics**						
Sex	0.288**	-0.032	0.033	0.216*	-0.014	0.155
Age	-0.064	0.037	0.145	0.065	0.123	0.054
Academic achievement	0.126	-0.002	-0.175*	0.030	0.048	0.013
Paternal education level	-0.049	-0.016	-0.115	-0.121	-0.098	-0.094
Maternal education level	0.069	-0.008	-0.022	-0.019	-0.070	0.011
Family socioeconomic status	-0.003	0.050	0.031	-0.047	0.051	0.047
**Clinical characteristics**						
Age of onset	0.078	-0.011	0.075	0.048	0.097	0.083
Duration of disease (months)	-0.077	0.065	0.076	0.141	0.117	0.044
Frequency of depressive episodes	0.015	0.017	0.098	0.185*	-0.023	0.116
Psychotic symptoms	0.048	-0.038	-0.043	0.008	-0.056	-0.018
Manic/hypomania	0.062	0.036	0.023	0.062	0.057	0.098
Severity of depression	0.275**	-0.008	0.044	0.151	0.046	0.156
Family history of mental disorders	-0.097	-0.080	0.195*	-0.107	-0.148	-0.162
Antidepressant treatment	-0.020	-0.047	-0.022	-0.065	-0.035	-0.044
**Clinical symptoms**						
SDS total score	0.629**	0.286**	0.216*	0.603**	0.174*	0.557**
SAS total score	0.672**	0.407**	0.303**	0.625**	0.336**	0.682**
**Family adaptability and cohesion**						
Family intimacy	-0.347**	-0.292**	-0.280*	-0.386**	-0.199*	-0.386**
Family adaptability	-0.393**	-0.256**	-0.215*	-0.415**	-0.139	-0.390**
**Parenting styles**						
Paternal emotional warmth and understanding	-0.237**	-0.226**	-0.142	-0.340**	-0.025	-0.279**
Maternal emotional warmth and understanding	-0.269**	-0.127	-0.164	-0.246**	-0.090	-0.252**
Paternal severe punishment	0.134	0.300**	0.256*	0.173**	0.104	0.261**
Maternal excessive interference	0.313**	0.314**	0.211*	0.195*	0.278**	0.357**
Paternal excessive interference	0.125	0.186*	0.154	0.004	0.112	0.148
Maternal refusal and denial	0.208*	0.298**	0.262**	0.220*	0.269**	0.336**
Paternal preference for subjects	-0.221**	-0.143	-0.071	-0.118	-0.138	-0.199*
Maternal severe punishment	0.289**	0.258**	0.264**	0.195*	0.218*	0.330**
Paternal refusal and denial	0.177*	0.308**	0.244**	0.139	0.186*	0.266**
Maternal preference for subjects	-0.211*	-0.072	-0.076	-0.124	-0.065	-0.155
Paternal overprotection	0.106	0.194*	0.191*	0.033	0.214*	0.201*
**Cognitive emotional regulation strategy**						
Self-blame	0.482**	0.133	0.275**	0.221**	0.175*	0.397**
Catastrophizing	0.527**	0.319**	0.228**	0.448**	0.367**	0.511**
Blaming of others	0.082	0.378**	0.094	0.168	0.241**	0.228**
Acceptance	0.045	-0.061	0.010	-0.152	0.030	-0.001
Rumination	0.325**	0.155	0.138	0.288**	0.138	0.297**
Positive refocusing	-0.330**	0.002	-0.023	-0.296**	0.046	-0.213*
Planning	-0.026	-0.016	-0.122	-0.142	-0.003	-0.075
Positive reappraisal	-0.336**	-0.086	0.011	-0.422**	-0.004	-0.266**
Putting into perspective	-0.254*	-0.214*	-0.210*	-0.260*	-0.184*	-0.203*
**Childhood trauma**						
Emotional abuse	0.449**	0.405**	0.288**	0.435**	0.183*	0.501**
Emotional neglect	0.434**	0.373**	0.279**	0.399**	0.145	0.476**
Physical abuse	0.193*	0.226**	0.196*	0.118	0.040	0.238**
Physical neglect	0.379**	0.440**	0.332**	0.384**	0.213*	0.504**
Sexual abuse	0.178*	0.182*	0.133	0.100	0.145	0.197*
CTQ total score	0.494**	0.441**	0.309**	0.436**	0.191*	0.547**

Note: * means *P* < 0.05; ** means *P* < 0.01

**Table 4.3:** Linear regression model of risk factors for overall HRRBs in adolescents with depression

Predictive variable	Linear regression coefficient	Standardized linear regression coefficient	T value	P
(Constants)	3.665		0.236	0.814
**Clinical symptoms**				
SDS total score	5.129	0.048	0.375	0.708
SAS total score	0.233	0.246	2.122	**0.036**
**Family adaptability and cohesion**				
Family intimacy	-0.262	-0.222	-1.598	0.113
Family adaptability	0.257	0.180	1.219	0.226
**Parenting styles**				
Paternal emotional warmth and understanding	-0.175	-0.133	-1.224	0.224
Maternal emotional warmth and understanding	0.301	0.224	2.071	**0.041**
Paternal severe punishment	-0.058	-0.029	-0.239	0.811
Maternal excessive interference	0.126	0.075	0.800	0.425
Maternal refusal and denial	0.544	0.191	1.399	0.165
Paternal preference for subjects	-0.229	-0.045	-0.636	0.526
Maternal severe punishment	-0.258	-0.091	-0.755	0.452
Paternal refusal and denial	-0.159	-0.038	-0.329	0.742
Paternal overprotection	0.460	0.090	0.915	0.362
**Cognitive emotional regulation strategy**				
Self-blame	0.480	0.098	1.218	0.226
Catastrophizing	0.927	0.282	3.148	**0.002**
Blaming of others	0.212	0.059	0.797	0.427
Rumination	-0.306	-0.077	-0.988	0.326
Positive refocusing	-0.092	-0.022	-0.264	0.792
Positive reappraisal	0.221	0.060	0.653	0.515
Putting into perspective	0.645	0.128	1.837	0.069
**Childhood trauma**				
Emotional abuse	0.616	0.163	0.978	0.330
Physical abuse	-0.223	-0.046	-0.414	0.680
Sexual abuse	0.756	0.093	1.128	0.262
Physical neglect	1.171	0.239	1.887	0.062
CTQ total score	-0.142	-0.120	-0.422	0.674

**Table 4.4:** Linear regression model of risk factors for SS in adolescents with depression

Predictive variable	Linear regression coefficient	Standardized linear regression coefficient	T value	P
(Constants)	-7.58		-1.558	0.122
**Demographic characteristics**				
Sex	0.573	0.048	0.737	0.463
**Clinical characteristics**				
Severity of depression	0.264	0.04	0.623	0.534
**Family adaptability and cohesion**				
Family intimacy	0.015	0.037	0.287	0.775
Family adaptability	-0.017	-0.035	-0.262	0.794
**Parenting styles**				
Paternal emotional warmth and understanding	-0.017	-0.039	-0.409	0.684
Maternal emotional warmth and understanding	-0.007	-0.014	-0.139	0.89
Maternal excessive interference	0.009	0.015	0.194	0.846
Maternal refusal and denial	-0.084	-0.086	-0.696	0.488
Paternal preference of subjects	-0.033	-0.019	-0.122	0.903
Maternal severe punishment	0.037	0.039	0.358	0.721
Paternal refusal and denial	0.092	0.065	0.728	0.468
Maternal preference for subjects	-0.046	-0.029	-0.183	0.855
**Clinical symptoms**				
SDS total score	6.363	0.176	1.475	0.143
SAS total score	0.089	0.277	2.601	**0.011**
**Cognitive emotional regulation strategy**				
Self-blame	0.368	0.221	2.924	**0.004**
Rumination	-0.062	-0.046	-0.632	0.529
Positive refocusing	-0.131	-0.094	-1.225	0.223
Positive reappraisal	0.069	0.056	0.632	0.529
Putting into perspective	0.141	0.082	1.242	0.217
Catastrophizing	0.215	0.192	2.415	**0.017**
**Childhood trauma**				
Emotional abuse	0.045	0.035	0.34	0.735
Physical abuse	-0.033	-0.02	-0.265	0.792
Sexual abuse	0.54	0.196	3.082	**0.003**
Emotional neglect	0.01	0.01	0.089	0.929
Physical neglect	-0.018	-0.011	-0.138	0.891

**Table 4.5:** Multiple linear regression model of risk factors for aggression and violent behavior in adolescents with depression

Predictive variable	Linear regression coefficient	Standardized linear regression coefficient	T value	P
(Constants)	4.295		0.781	0.436
**Family adaptability and cohesion**				
Family intimacy	-0.125	-0.334	-2.082	**0.04**
Family adaptability	0.16	0.354	2.181	**0.031**
**Parenting styles**				
Paternal emotional warmth and understanding	0.018	0.042	0.372	0.71
Paternal severe punishment	0.035	0.054	0.381	0.704
Maternal excessive interference	0.075	0.141	1.204	0.231
Paternal excessive interference	-0.102	-0.111	-0.982	0.328
Maternal refusal and denial	0.017	0.019	0.121	0.904
Maternal severe punishment	-0.161	-0.179	-1.278	0.204
Paternal refusal and denial	0.09	0.069	0.501	0.618
Paternal overprotection	0.13	0.08	0.686	0.494
**Clinical symptoms**				
SDS total score	-4.386	-0.131	-0.968	0.335
SAS total score	0.044	0.145	1.09	0.278
**Cognitive emotional regulation strategy**				
Putting into perspective	0.13	0.081	1.013	0.313
Catastrophizing	0.149	0.143	1.495	0.138
Blaming of others	0.235	0.207	2.476	**0.015**
**Childhood questionnaire**				
Emotional abuse	0.41	0.344	1.743	0.084
Physical abuse	0.079	0.051	0.399	0.691
Sexual abuse	0.121	0.047	0.484	0.629
Physical neglect	0.527	0.34	2.291	**0.024**
CTQ total score	-0.102	-0.272	-0.82	0.414

**Table 4.6:** Linear regression model of risk factors for health-compromising behavior in adolescents with depression

Predictive variable	Linear regression coefficient	Standardized linear regression coefficient	T value	P
(Constants)	3.486		1.019	0.31
**Demographic characteristics**				
Sex	0.29	0.036	0.536	0.593
**Clinical characteristics**				
Frequency of depressive episodes	0.833	0.154	2.322	**0.022**
**Family adaptability and cohesion**				
Family intimacy	0.007	0.027	0.203	0.839
Family adaptability	-0.035	-0.105	-0.763	0.447
**Parenting styles**				
Paternal emotional warmth and understanding	-0.079	-0.26	-2.758	**0.007**
Maternal emotional warmth and understanding	0.067	0.216	2.129	**0.035**
Paternal severe punishment	-0.04	-0.085	-0.923	0.358
Maternal excessive interference	0.006	0.014	0.183	0.855
Maternal refusal and denial	0.137	0.207	1.845	0.068
Maternal severe punishment	-0.122	-0.186	-1.683	0.095
**Clinical symptoms**				
SDS total score	3.703	0.151	1.196	0.234
SAS total score	0.058	0.266	2.405	**0.018**
**Cognitive emotional regulation strategy**				
Self-blame	-0.006	-0.005	-0.073	0.942
Positive refocusing	-0.06	-0.063	-0.807	0.421
Positive reappraisal	-0.085	-0.101	-1.116	0.267
Putting into perspective	-0.106	-0.135	-2.053	0.117
Catastrophizing	0.121	0.159	2.139	**0.035**
**Childhood trauma**				
Emotional abuse	0.015	0.017	0.108	0.914
Emotional neglect	-0.05	-0.073	-0.476	0.635
Physical neglect	0.108	0.096	0.812	0.419
CTQ total score	0.015	0.055	0.199	0.843

## Abbreviations

HRRBs: health-related risky behaviors
AHRBI: Adolescent Health-Related Risky Behavior Inventory
SAS: Self-Rating Anxiety Scale
SDS: Self-Rating Depression Scale
CTQ: Childhood Trauma Questionnaire
CERQ: Cognitive Emotion Regulation Questionnaire
EMBU: Egna Minnen av Barndoms Uppfostran
FACES II-CV: Family Adaptability and Cohesion Scale
SS: self-injury and suicide
AV: aggression and violence
RB: rule-breaking
SD: smoking and drinking
HCB: health-compromising behavior
